# Proline Potentiates Aminoglycoside Bactericidal Efficacy Against *Staphylococcus aureus*

**DOI:** 10.3390/life16071070

**Published:** 2026-06-26

**Authors:** Bo-Hao Li, Rui-Hua Xu, Zulifukeer Maituersong, Chao-Feng Lai, Ting Wang, Yu-Bin Su

**Affiliations:** 1 Department of Immunology and Microbiology & Institute of Medical Microbiology, College of Life Science and Technology, Jinan University, Guangzhou 510632, China; 2 MOE Key Laboratory of Viral Pathogenesis & Infection Prevention and Control (Jinan University), National Engineering Research Center of Genetic Medicine, Guangdong Provincial Key Laboratory of Bioengineering Medicine, College of Life Science and Technology, Jinan University, Guangzhou 510632, China; 3 Department of Biological Sciences and Biotechnology, College of Life Science and Technology, Jinan University, Guangzhou 510632, China

**Keywords:** *Staphylococcus aureus*, zoonotic pathogen, antibiotic resistance, amikacin, PMF, ROS, NO

## Abstract

*Staphylococcus aureus* is an important zoonotic pathogen. In recent years, it has been isolated from diseased aquatic animals, causing skin ulcers and septicemia, establishing itself as an emerging pathogen in aquaculture. Rampant antibiotic use has accelerated antimicrobial resistance, a trend that has gradually curtailed the potency of conventional antibiotic therapies, underscoring the urgent need for novel therapies. Here, we screened 20 amino acids and found that exogenous proline significantly enhances the bactericidal activity of amikacin against *S. aureus*. This synergistic effect extends to other aminoglycoside antibiotics, including neomycin sulfate and gentamicin, and is also effective against drug-resistant strains such as MRSA USA300. Furthermore, we evaluated the efficacy of this combination in eradicating persisters and biofilms. Mechanistically, exogenous proline potentiates amikacin-mediated killing by modulating two key bactericidal pathways. On one hand, it enhances antibiotic uptake by augmenting the proton motive force via the electron transport chain. On the other hand, it amplifies oxidative stress through a multi-pronged mechanism involving the suppression of ROS-scavenging enzymes, activation of the Fenton reaction, and reduction in intracellular nitric oxide (NO) levels, ultimately culminating in bacterial cell death. This study proposes a promising strategy for combating *S. aureus* in aquaculture and healthcare-associated infections.

## 1. Introduction

The misuse and overuse of antibiotics in human medicine have accelerated the spread of drug-resistant bacteria, driving the continuous emergence of multidrug-resistant (MDR) and extensively drug-resistant (XDR) pathogens in clinical settings [1,2]. This trend severely undermines the efficacy of conventional therapies, making common infections increasingly difficult—and sometimes impossible—to treat, thereby posing a serious global public health threat. Without effective intervention, antimicrobial resistance (AMR) is projected to become the leading cause of death worldwide by 2050, responsible for an estimated 10 million annual fatalities [3]. Consequently, exploring old drug repurposing strategies, identifying new antimicrobial targets, and developing innovative therapeutic strategies are now critical priorities in biomedical and public health research.

Aquaculture is one of the fastest-growing food production sectors globally, with its output reaching 94 million tonnes of seafood and 35 million tonnes of aquatic plants in 2022 [4]. To manage disease outbreaks in high-density farming, antibiotics have been extensively used—particularly in Asian countries—thereby accelerating resistance evolution in aquatic pathogens. Widespread overuse of antibiotics in aquaculture leads to roughly 80% of active antimicrobials being released into the environment (surface water, sediment) and drives the emergence of resistant bacteria [5]. The resultant antibiotic residues and disseminated drug-resistant bacteria threaten animal health, food safety and public health via zoonotic transmission, forming an “aquaculture environment–animal–human” resistance cycle. Therefore, developing strategies to reduce antibiotic usage while effectively treating *S. aureus* infections is of great importance for ensuring aquaculture safety, food safety, and public health.

*Staphylococcus aureus*, traditionally considered a human pathogen, has in recent years been isolated and identified from diseased aquatic organisms, including tilapia, sea bream, and shrimp, where it causes skin ulcers, hemorrhagic septicemia, and high mortality, establishing itself as an emerging aquatic pathogen [6,7]. *S. aureus* is also commonly found in the environment (including surface water, sediment) and on human and animal skin and mucosa [8,9]. Contact transmission can lead to diverse infections, including skin and soft tissue infections, pneumonia, sepsis, and endocarditis, which may progress to organ failure and death. Of particular concern are drug-resistant variants such as methicillin-resistant *S. aureus* (MRSA), which demonstrate high transmissibility, enhanced virulence, and multi-drug resistance, presenting major challenges in clinical management [10,11]. Notably, the pathogenicity of *S. aureus* in aquaculture has received increasing attention. Isolates from diseased tilapia can produce hemolysins and form biofilms, causing severe skin lesions and systemic infections. In intensive aquaculture systems, bacteria can form biofilms on water, pond walls, and fish mucosal surfaces, increasing the difficulty of disinfection and the risk of disease transmission [12]. Moreover, aquaculture-derived *S. aureus* isolates often carry multidrug resistance genes and exhibit resistance to multiple antibiotics, including aminoglycosides, thereby limiting treatment options. A survey from commercial aquaculture farms in Egypt revealed that 50% of *S. aureus* isolates were MRSA, carrying the *icaA*/*icaD* biofilm-forming genes and various virulence factors [6]. Further studies in Nigeria confirmed cross-transmission of *S. aureus* among fish, water, and aquaculture workers, indicating a significant zoonotic risk [13].

Current treatment of *S. aureus* infections in humans relies mainly on β-lactams and glycopeptides, with oxazolidinones and macrolides as alternative options [14]. Specifically, oxacillin and cloxacillin are first-line for methicillin-susceptible *S. aureus* (MSSA), whereas vancomycin and teicoplanin are cornerstone therapies for MRSA [14,15]. Additionally, many studies have evaluated the bactericidal effect of aminoglycoside-based combinations against *S. aureus*, including but not limited to chenodeoxycholic acid with amikacin, fatty acids with aminoglycosides, and amikacin with levofloxacin [16,17,18]. Aminoglycosides exert their bactericidal effect by binding to the 30S ribosomal subunit and interfering with protein synthesis. Gentamicin and neomycin are aminoglycosides with a history of application in aquaculture [19]. Amikacin is a semi-synthetic derivative of kanamycin A, obtained by introducing an L-γ-amino-α-hydroxybutyryl side chain at the C-1 position of the 2-deoxystreptamine ring, and is currently the most widely used semi-synthetic aminoglycoside antibiotic in both clinical and research settings [20]. Due to its structural modification, amikacin is resistant to most aminoglycoside-modifying enzymes, including phosphotransferases, nucleotidyltransferases, and the majority of acetyltransferases, thereby exhibiting a broader antimicrobial spectrum [20], which prompted us to select amikacin for the present investigation.

Exogenous supplementation of bacterial endogenous metabolites can reverse antibiotic resistance and restore bacterial susceptibility by modulating the bacteria’s intrinsic metabolic and drug resistance mechanisms. Fumarate, indole, and various amino acids have been shown to enhance the tricarboxylic acid (TCA) cycle or the pyruvate cycle, thereby reversing drug resistance [21,22,23]. Sugar metabolites exhibit similar effects; for example, galactose reverses meropenem resistance in *Salmonella enteritidis* [24]. Notably, pyruvate combined with gentamicin effectively kills drug-resistant *Vibrio alginolyticus* [25], a major pathogen in marine aquaculture. This finding provides direct evidence for the feasibility of metabolite-assisted strategies in controlling aquatic pathogens and suggests that similar metabolite-antibiotic synergy may be applicable to other aquaculture-associated bacteria, including Gram-positive *S. aureus*. Furthermore, metabolic intervention can reverse drug resistance through other mechanisms. For instance, bacteria can regulate resource allocation through metabolism-driven post-translational modifications of proteins, thereby altering their susceptibility to antibiotics [26,27]. Natural compounds such as paeonol have been shown to enhance the antimicrobial activity against *Aeromonas hydrophila* [28], a common opportunistic pathogen in freshwater aquaculture, by modulating efflux pump activity and membrane permeability. Collectively, these studies indicate that targeting metabolic pathways represents a novel perspective for understanding and addressing bacterial drug resistance and provides a theoretical foundation for developing exogenous metabolite supplementation strategies applicable to aquaculture environments.

Amino acids serve not only as protein building blocks but also as key regulators of bacterial metabolism, stress adaptation, virulence, and homeostasis [29,30]. They support growth, maintain redox and metabolic balance, and aid in stress responses [31]. Under nutrient limitation, bacteria maintain amino acid pools through synthesis and uptake, with some amino acids acting as stress protectants [32]. In aquatic nutrition, the functions of amino acids extend beyond protein synthesis. Various amino acids have been widely used in aquafeeds as functional additives, playing multiple roles. Glutamine and arginine enhance immune responses and intestinal health in fish. Proline, glycine, and alanine function as osmoprotectants, helping fish cope with environmental stresses such as salinity and temperature fluctuations while maintaining cellular osmotic balance. Additionally, arginine and proline are involved in collagen synthesis, promoting tissue repair and wound healing [33]. These applications indicate that exogenous amino acids can enhance disease resistance in farmed aquatic animals by modulating their physiological state. However, the strategy of using amino acids as antibiotic adjuvants to directly intervene in drug resistance of aquatic pathogens remains to be further explored. Recent studies have demonstrated that various amino acids exert synergistic bactericidal effects when combined with antibiotics. Several amino acids—including arginine, and alanine—synergize with antibiotics to enhance killing of resistant bacteria [34,35]. An amino acid formulation (18AA) also boosts the activity of ceftazidime–avibactam against MDR pathogens [36]. Other pathways, such as the pyruvate and ubiquitin systems, further intersect with amino acid metabolism to modulate drug susceptibility [37,38], underscoring the important role of amino acids in reversing resistance.

In the present study, we hypothesized that exogenous amino acid supplementation could potentiate antibacterial activity against *S. aureus* by modulating bacterial metabolic pathways. To test this hypothesis, we conducted the following investigations: 1. screening proteinogenic amino acids for synergistic activity with diverse antimicrobial agents; 2. evaluating the efficacy of the optimal combination against clinically recalcitrant persisters and biofilms—two major therapeutic challenges [39,40]; 3. elucidating the underlying mechanisms involving proton motive force, reactive oxygen species, and nitric oxide. This work provides a foundation for developing antibiotic-sparing, old drug repurposing strategies to combat *S. aureus* infections in both aquaculture and clinical settings.

## 2. Materials and Methods

### 2.1. Instruments

Vertical autoclave (Dengguan Medical, Changzhou, China); constant-temperature shaking incubator (Taicang Huamei Laboratory Instrument Factory, Taicang, China); ultra-clean workbench (Suzhou Purifying Equipment Co., Ltd., Suzhou, China); Model 721 visible spectrophotometer (Shanghai Youke Instrument Co., Ltd., Shanghai, China); refrigerated centrifuge (Thermo Fisher Scientific (China) Co., Ltd., Shanghai, China); ultrasonic cell disruptor & ice maker (Ningbo Xinzhi Biotechnology Co., Ltd., Ningbo, China); constant-temperature drying oven (Shanghai Yiheng Scientific Instrument Co., Ltd., Hangzhou, China); pipettes (Eppendorf (Shanghai), Shanghai, China); analytical balance (Shanghai Yingheng Weighing Equipment Co., Ltd., Shanghai, China); ultra-low-temperature refrigerator (Haier, Qingdao, China); microwave oven (Midea Group Co., Ltd., Foshan, China); GLARIOstar PLUS multimode microplate reader (BMG, Ortenberg, Baden-Württemberg, Germany); flow cytometer (Beckman Coulter, Brea, CA, USA).

### 2.2. Reagents

#### 2.2.1. Culture Media

Luria–Bertani (LB) medium components (Bacto-peptone, yeast extract) (Guangzhou Huankai Biotechnology Co., Ltd., Guangzhou, China); sodium chloride (NaCl) (Macklin Biochemical Co., Ltd., Shanghai, China); agar powder, sodium hydroxide, sodium acetate, magnesium sulfate, calcium chloride, disodium hydrogen phosphate dodecahydrate, dipotassium hydrogen phosphate, ammonium chloride (Sangon Biotech (Shanghai) Co., Ltd., Shanghai, China).

#### 2.2.2. Metabolites and Inhibitors

Alanine, arginine, asparagine, aspartic acid, cysteine, glutamine, glutamic acid, glycine, histidine, isoleucine, leucine, lysine, methionine, phenylalanine, proline, serine, threonine, tryptophan, tyrosine, valine (Macklin Biochemical Co., Ltd., Shanghai, China); malonic acid (Yuanye Bio-Technology, Shanghai, China); sodium azide (Macklin Biochemical Co., Ltd., Shanghai, China); S-nitrosoglutathione (Shanghai Dibai Biotechnology Co., Ltd., Shanghai, China); CCCP (Sigma-Aldrich, St. Louis, MO, USA).

#### 2.2.3. Antibiotics

Gentamicin, moxifloxacin (Shanghai Aladdin Biochemical Technology Co., Ltd., Shanghai, China); tobramycin, ribostamycin, streptomycin, kanamycin, amikacin, neomycin, cefazolin, doxycycline, vancomycin (Macklin Biochemical Co., Ltd., Shanghai, China). 

### 2.3. Bacterial Strains and Culture Conditions

The bacterial strains utilized in this study were *S. aureus* ATCC6538, *S. aureus* USA300_FPR3757, and *Corynebacterium diphtheriae*. All strains were stored as glycerol stocks at −80 °C. Stock cultures maintained on agar plates were stored at 4 °C. For each experiment, bacterial cultures were grown in LB liquid medium at 37 °C with shaking at 220 rpm for 12 h (24 h for *C. diphtheriae*). All experiments were performed in three independent biological replicates. 

### 2.4. Antibiotic Killing Assay

Overnight bacterial cultures (12 h) grown in fresh LB medium were harvested, washed three times with sterile saline, and resuspended in M9 medium (supplemented with 10 mM acetate, 1 mM MgSO_4_, and 100 µM CaCl_2_) to an optical density at 600 nm (OD600) of 0.2. Bacterial suspension was incubated at 37 °C with shaking at 220 rpm for 6 h in the presence or absence of 300 μg/mL amikacin and/or 5 mM proline. After incubation, 100 μL aliquots of the cultures were serially diluted, and 10 μL of each dilution was spotted onto LB agar plates. Plates were incubated at 37 °C for 12 h, and colonies were counted to determine colony-forming units (CFU/mL). 

### 2.5. Isolation of Bacterial Persisters

*S. aureus* persisters were generated according to established protocols [39,40]. Bacteria were cultured as described above (see Bacteria Culture Conditions). The overnight culture was transferred to sterile 50 mL centrifuge tubes and centrifuged at 8000× *g* for 5 min at room temperature. The cell pellet was collected, washed three times with sterile saline, and resuspended in 25% brain heart infusion (BHI)-M9 medium to an OD600 of 0.2. To eliminate non-persister cells, moxifloxacin was added to a final concentration of 100 µg/mL, and the culture was incubated for an additional 3 h. Persisters were subsequently collected by centrifugation and washed to remove residual antibiotic.

### 2.6. Biofilm Formation Inhibition Assay

The biofilm formation inhibition assay was conducted using crystal violet staining to quantify biofilm biomass. Bacterial precultures were prepared following the bacterial viability measurement protocol. Bacteria were harvested by centrifugation and resuspended in M9 minimal medium to an OD_600_ of 0.2. Aliquots of 3 mL bacterial suspension were dispensed into test tubes and treated with or without 5 mM proline plus 300 μg/mL amikacin. A volume of 200 μL treated bacterial suspension was transferred into each well of a 96-well microplate, followed by static incubation at 37 °C for 6 h. After incubation, the M9 medium was discarded, and wells were washed twice with sterile saline before air-drying at room temperature. Each well was stained with 150 μL of 0.1% crystal violet solution for 20 min. Excess unbound crystal violet was removed by washing with sterile saline, and plates were air-dried again. A total of 150 μL of 95% ethanol was added to each well to solubilize the bound crystal violet, and plates were mixed thoroughly by shaking. The absorbance of each well was measured at 570 nm using a multimode microplate reader.

### 2.7. In Vitro Biofilm Model

Biofilms were prepared according to established protocol [41]. Bacteria were cultured as described above (see Bacteria Culture Conditions). A 10 μL aliquot of the overnight culture was transferred into a 6 mm PE50 catheter tube (0.58 mm × 0.96 mm, inner diameter × outer diameter), which was then placed into a 1.5 mL microcentrifuge tube containing 1 mL of fresh LB broth and incubated at 37 °C. Three biological replicates were prepared per treatment group. The LB medium was replaced with fresh medium every 24 h, and the cultures were maintained for 3 days to allow biofilm maturation. Following incubation, each catheter was gently rinsed three times with sterile saline to remove non-adherent bacteria. Catheters with established biofilms were subsequently used in biofilm eradication assays. The biofilm preparation protocol demonstrated relatively robust reproducibility, with biomass measurements from six biological replicates all approximating 2.4 × 10^7^ CFU/cm^2^. 

### 2.8. Measurement of NADH Levels

Intracellular NADH levels were quantified using a commercial NADH assay kit (S0175, Beyotime, Shanghai, China). Bacterial cells were harvested, resuspended in M9 medium to an OD600 of 0.2, and incubated at 37 °C with shaking (220 rpm) for 6 h in the presence or absence of 300 µg/mL amikacin and/or 5 mM proline. Following incubation, 10 mL aliquots of each culture were centrifuged at 8000× *g* for 5 min. The cell pellets were resuspended in PBS and subjected to sonication on ice for 15 min (200 W total power, 50% duty cycle, 2 s on/3 s off pulses). The lysates were then centrifuged at 12,000× *g* for 10 min at 4 °C, and the resulting supernatants were analyzed for NADH content following the manufacturer’s protocol. 

### 2.9. Detection of ATP

Intracellular ATP levels were determined using the BacTiter-Glo™ Microbial Cell Viability Assay (Promega, Mannheim, Germany) [42]. Overnight bacterial cultures were harvested, washed with sterile saline, and resuspended in 5 mL of M9 medium to an OD600 of 0.2. The suspensions were incubated at 37 °C with shaking (220 rpm) for 6 h in the presence or absence of 300 µg/mL amikacin and/or 5 mM proline. Following incubation, 50 µL aliquots of each culture were transferred to a 96-well plate, mixed with 50 µL of BacTiter-Glo reagent, and luminescence was immediately measured using a CLARIOstar microplate reader in accordance with the manufacturer’s instructions. 

### 2.10. Measurement of Membrane Potential

Bacterial membrane potential was assayed following a previously described protocol with slight modifications [43]. Overnight bacterial cultures were resuspended in M9 medium to an OD600 of 0.2 and incubated at 37 °C with shaking (220 rpm) for 6 h, treated with or without 300 µg/mL amikacin and/or 5 mM proline. After incubation, cells were diluted in PBS to approximately 1 × 10^6^ CFU/mL and stained with 10 µM DiOC_2_(3) at 37 °C for 30 min in the dark. The stained samples were then analyzed on a flow cytometer (Beckman Coulter, Brea, CA, USA), with 10,000 events recorded per sample. Forward scatter (FSC) and side scatter (SSC) thresholds were set at 8000. Briefly, an initial FSC-A vs. SSC-A dot plot was generated to construct a primary gate that excluded cellular debris and electronic noise, retaining intact bacterial particles. A secondary FSC-H vs. FSC-A gate was subsequently applied to discriminate single bacterial cells from doublets or cell aggregates. Only events passing both gating criteria were included for subsequent fluorescence analysis. Unstained bacterial suspensions prepared under identical culture conditions were run in parallel as negative controls to define baseline green and red fluorescence signals, and fluorescence quadrant regions were delineated based on the signal distribution of unstained and DiOC_2_(3)-stained single cells to evaluate the shift in red and green fluorescence intensity induced by different treatments. The red-to-green fluorescence ratio (mCherry/FITC) was measured for each cell and normalized. Membrane potential was calculated according to the following formula: Membrane Potential = log_10_((red fluorescence)/(green fluorescence) × 10^3/2^). 

### 2.11. Intracellular Amikacin Uptake Assay

Intracellular amikacin concentrations were quantified using a commercial amikacin ELISA rapid test kit (Huabo Deyi, Beijing, China). Overnight bacterial cultures were harvested and resuspended in M9 medium to an OD600 of 1.0. The suspensions were incubated at 37 °C with shaking (220 rpm) for 6 h in the presence of 300 µg/mL amikacin, with or without 5 mM proline. After incubation, bacterial cells were collected by centrifugation, washed three times with PBS to remove extracellular antibiotic, and lysed via sonication on ice for 15 min (200 W total power, 50% duty cycle, 2 s on/3 s off pulses). The lysates were centrifuged at 12,000× *g* for 10 min at 4 °C, and the resulting supernatants were collected and normalized to the total protein content of each sample as determined by the BCA protein assay. Amikacin concentration in the supernatants was determined following the manufacturer’s protocol for the ELISA kit. 

### 2.12. Measurement of Intracellular ROS

Intracellular reactive oxygen species (ROS) levels were assessed following a previously described protocol [44]. Overnight bacterial cultures were harvested, washed with sterile saline, and resuspended in 5 mL of M9 medium to an OD600 of 0.2. The suspensions were incubated at 37 °C with shaking (220 rpm) for 6 h in the presence or absence of 300 μg/mL amikacin and/or 5 mM proline. After incubation, 196 μL aliquots of each culture were transferred to a black 96-well microplate (Beijing Labgic Technology Co., Ltd., No.9 Yumin Street, Area B, Airport Industrial Zone, Shunyi District, Beijing, China) and mixed with 4 μL of 2’,7’-dichlorofluorescein diacetate (DCFH-DA; Sigma-Aldrich, St. Louis, MO, USA) to a final concentration of 20 μM. The plate was incubated at 37 °C in the dark for 30 min, and fluorescence intensity was immediately measured using a CLARIOstar microplate reader with excitation at 485 nm and emission at 515 nm. 

### 2.13. SOD Activity Assay

Superoxide dismutase (SOD) activity was quantified using a commercial SOD assay kit (Solarbio, Beijing, China). Overnight bacterial cultures were harvested and resuspended in M9 medium to an OD600 of 1.0. The suspensions were incubated at 37 °C with shaking (220 rpm) for 6 h in the presence or absence of 300 μg/mL amikacin and/or 5 mM proline. Following incubation, bacterial cells were collected by centrifugation, washed three times with PBS, and resuspended in PBS. Cell suspensions were lysed on ice by sonication for 15 min (200 W total power, 50% duty cycle, 2 s on/3 s off pulses). The lysates were centrifuged at 12,000× *g* for 10 min at 4 °C to remove debris, and the supernatants were collected. Protein concentrations in the supernatants were determined with a BCA protein assay kit. SOD activity was then measured according to the manufacturer’s protocol, and absorbance was recorded at 560 nm using a microplate reader. 

### 2.14. CAT Activity Assay

Catalase (CAT) activity was measured using a commercial assay kit (Nanjing Jiancheng, China). Overnight bacterial cultures were harvested and resuspended in M9 medium to an OD600 of 1.0. The suspensions were incubated at 37 °C with shaking (220 rpm) for 6 h in the presence or absence of 300 µg/mL amikacin and/or 5 mM proline. Following incubation, bacterial cells were collected by centrifugation, washed three times with PBS, and resuspended in PBS. The cell suspensions were lysed via sonication on ice for 15 min (200 W total power, 50% duty cycle, 2 s on/3 s off pulses). The lysates were centrifuged at 12,000× *g* for 10 min at 4 °C to remove cell debris, and the supernatants were collected. Protein concentrations in the supernatants were determined using a BCA protein assay kit. CAT activity was then measured according to the manufacturer’s protocol, and absorbance was recorded at 405 nm using a microplate reader. 

### 2.15. Quantification of Intracellular Ferrous Iron (Fe^2+^)

Intracellular Fe^2+^ levels were measured using a commercial iron assay kit (Solarbio, Beijing, China). Overnight bacterial cultures were harvested and resuspended in M9 medium to an OD600 of 1.0. The suspensions were incubated at 37 °C with shaking (220 rpm) for 6 h in the presence or absence of 300 μg/mL amikacin and/or 5 mM proline. After incubation, bacterial cells were collected by centrifugation at 8000× *g* for 5 min. The cell pellets were resuspended in PBS and lysed on ice by sonication for 15 min (200 W total power, 50% duty cycle, 2 s on/3 s off pulses). The lysates were centrifuged at 12,000× *g* for 10 min at 4 °C to remove cell debris, and the resulting supernatants were collected. Fe^2+^ concentrations in the supernatants were determined according to the manufacturer’s instructions. 

### 2.16. Measurement of Intracellular Nitric Oxide (NO)

NO levels were determined as previously described, with modifications [45]. Overnight bacterial cultures were harvested, washed three times with PBS, and resuspended in M9 medium to an OD600 of 0.2. The suspensions were incubated at 37 °C with shaking (220 rpm) for 6 h in the presence or absence of 300 μg/mL amikacin and/or 5 mM proline. Following incubation, bacterial cells were collected by centrifugation at 12,000× *g* for 10 min. The cell pellets were resuspended in PBS containing 5 μM DAF-FM DA (4-amino-5-methylamino-2’,7’-difluorofluorescein diacetate) and incubated at 37 °C in the dark for 30 min. After staining, cells were washed three times with PBS to remove excess probe. Fluorescence intensity was immediately measured using a CLARIOstar microplate reader with excitation at 495 nm and emission at 515 nm. 

### 2.17. Determination of MIC

The strains were activated with Mueller–Hinton broth (MHB) medium overnight, transferred to fresh MHB medium at a 1:100 volume ratio and incubated until OD600 = 0.5, followed by a 100-fold dilution of the bacterial solution. MHB medium and antibiotics were added to 96-well plates, and the antibiotics were sequentially diluted 2-fold, followed by 10 solution. The negative control group consisted of inoculated MHB medium only, and the µL of bacterial MIC was observed after incubation at 37 °C for 12 h. The minimal antibiotic concentrations that displayed no visible growth were determined as MIC values. 

### 2.18. Instrument Measurement Accuracy and Uncertainty

The constant-temperature shaking incubator (Taicang Huamei Laboratory Instrument Factory, Taicang, China) possessed a temperature control accuracy of ±0.1 °C and a temperature uniformity of ±0.5 °C. For the Model 721 visible spectrophotometer (Shanghai Youke Instrument Co., Ltd., Shanghai, China), the transmittance accuracy was ±1% T, and the photometric stability was ±0.004 A/h at 500 nm. The GLARIOstar PLUS multimode microplate reader (BMG, Ortenberg, Baden-Württemberg, Germany) exhibited the following photometric performance specifications for absorbance (ABS) measurements: absorbance range of 0–4 OD; photometric accuracy < 1% at 2 OD; photometric precision < 0.5% at 1 OD and <0.8% at 2 OD.

## 3. Results

### 3.1. Exogenous Proline Potentiates the Killing of S. aureus by Amikacin

**Figure 1:**
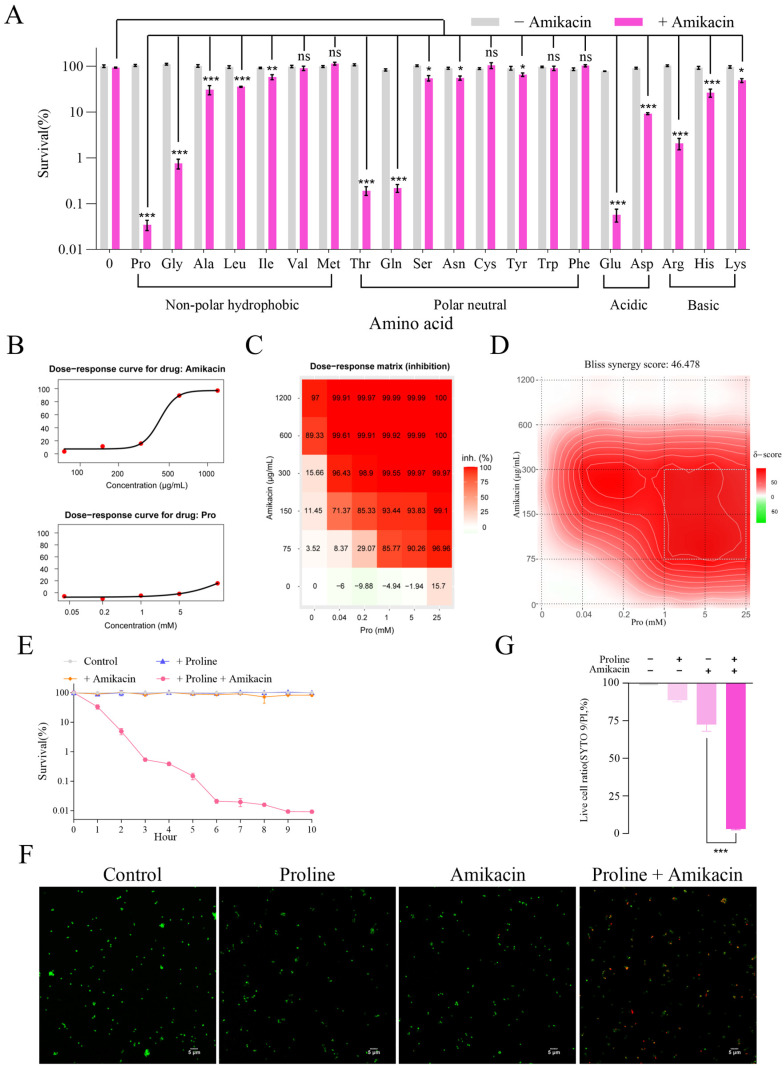
Exogenous proline enhances the bactericidal activity of amikacin against *S. aureus*. (**A**) Survival of *S. aureus* ATCC6538 treated with 300 μg/mL amikacin in combination with various amino acids (5 mM each). (**B**) Dose–response curves for amikacin and proline as single agents. (**C**) Dose–response matrix for the combination of amikacin and proline (inhibition rate). (**D**) Bliss synergy score contour plot for the combination of amikacin and proline. Red regions (δ-score > 0) indicate synergy, while green regions (δ-score < 0) indicate antagonism. (**E**) Time-dependent killing of bacteria treated with or without 300 µg/mL amikacin and/or 5 mM proline. (**F**) Representative confocal microscopy images stained with the Live/Dead Bacterial Viability Kit. Bacteria were treated with 5 mM proline, 300 µg/mL amikacin, or the combination. Green fluorescence (DMAO) indicates viable bacteria; red fluorescence (EthD-III) indicates dead bacteria. (**G**) Quantitative analysis of confocal microscopy images. Bacteria were stained with the Live/Dead Bacterial Viability Kit and imaged by confocal microscopy. All data were presented as the mean ± SEM (*n* = 3). Statistical significance was determined by one-way ANOVA. (ns means no significant difference; * *p* < 0.05, ** *p* < 0.01, *** *p* < 0.001).

To identify effective antibiotic combinations, we screened 20 amino acids with various antibiotics against *S. aureus* ATCC6538. Bactericidal assays were performed in M9 medium (with strong acid-base buffering capacity), with all 20 amino acids supplemented at 5 mM to maintain comparable pH and osmolarity across groups. Amikacin showed the strongest synergy with exogenous amino acids, particularly with proline, which achieved the highest killing efficacy (Figure 1A and Figure S1A–C). To screen for the optimal concentration combination, we employed the Bliss independence model to evaluate various concentration gradient combinations, which identified 300 μg/mL amikacin plus 5 mM proline as the most potent pairing with the highest synergy score of 82.58 and bactericidal efficacy exceeding that of amikacin alone by over 2000-fold (Figure 1B–D). Further, we conducted time-kill synergy studies on this combination and found that the bactericidal effect plateaued after 6 h (Figure 1E). Fluorescence microscopy confirmed the synergy, showing a marked shift from green (viable) fluorescence in single-agent treatments to orange/red fluorescence (indicating membrane damage and cell death) in the combination group, and live/dead cell counting analysis further showed that the proportion of dead cells in the combination group was significantly higher than that in the amikacin-alone group (Figure 1F,G).

### 3.2. Exogenous Proline Eliminates Persisters and Biofilms of S. aureus with Amikacin

**Figure 2:**
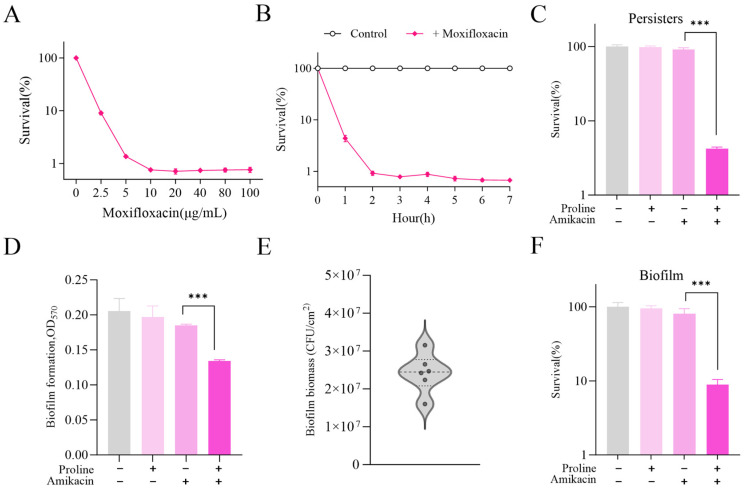
Exogenous proline enhances the bactericidal activity of amikacin against *S. aureus* persisters and biofilms. (**A**) Bactericidal activity of moxifloxacin against *S. aureus* ATCC6538 at various concentrations for the purpose of identifying the optimal concentration for persister induction. (**B**) Time-dependent killing of *S. aureus* ATCC6538 treated with moxifloxacin (100 µg/mL) for the purpose of identifying the optimal treatment time for persister induction. (**C**) Survival of bacterial persisters treated with 300 µg/mL amikacin and/or 5 mM proline. (**D**) Inhibition of biofilm formation by bacteria treated with or without 300 µg/mL amikacin and/or 5 mM proline. Biofilm biomass was quantified by crystal violet staining and measured at OD_570_. (**E**) Biofilm viable cell counts were determined by CFU assay and expressed as CFU/cm^2^. Each dot represents an independent replicate. The solid line indicates the median, and dashed lines represent the interquartile range, *n* = 6. (**F**) Survival of preformed biofilms treated with 300 µg/mL amikacin and/or 5 mM proline. All data were presented as the mean ± SEM (*n* = 3). Statistical significance was determined by one-way ANOVA (*** *p* < 0.001).

To evaluate the efficacy of the proline and amikacin combination against bacterial persisters, we used a moxifloxacin-induced persister model of *S. aureus* ATCC6538 [46,47] (Figure 2A,B). Bactericidal assays showed that exogenous proline significantly enhanced amikacin-mediated killing of these persisters (Figure 2C). We also evaluated the inhibitory effect of this combination on biofilm formation and found that the combination group significantly suppressed biofilm formation (Figure 2D). Simultaneously, we established preformed biofilms using 6 mm PE50 catheters with an average biomass of approximately 2.4 × 10^7^ CFU/cm^2^, and treated them with proline and amikacin, finding that this combination also significantly eradicated the established biofilms (Figure 2E,F). These results indicate that the proline/amikacin combination effectively eliminates persisters, inhibits biofilm formation, and eradicates established biofilms, underscoring its therapeutic potential against persistent and biofilm-associated infections in both clinical and aquaculture settings.

### 3.3. Exogenous Proline Increases Amikacin Uptake by Enhancing Proton Motive Force

**Figure 3:**
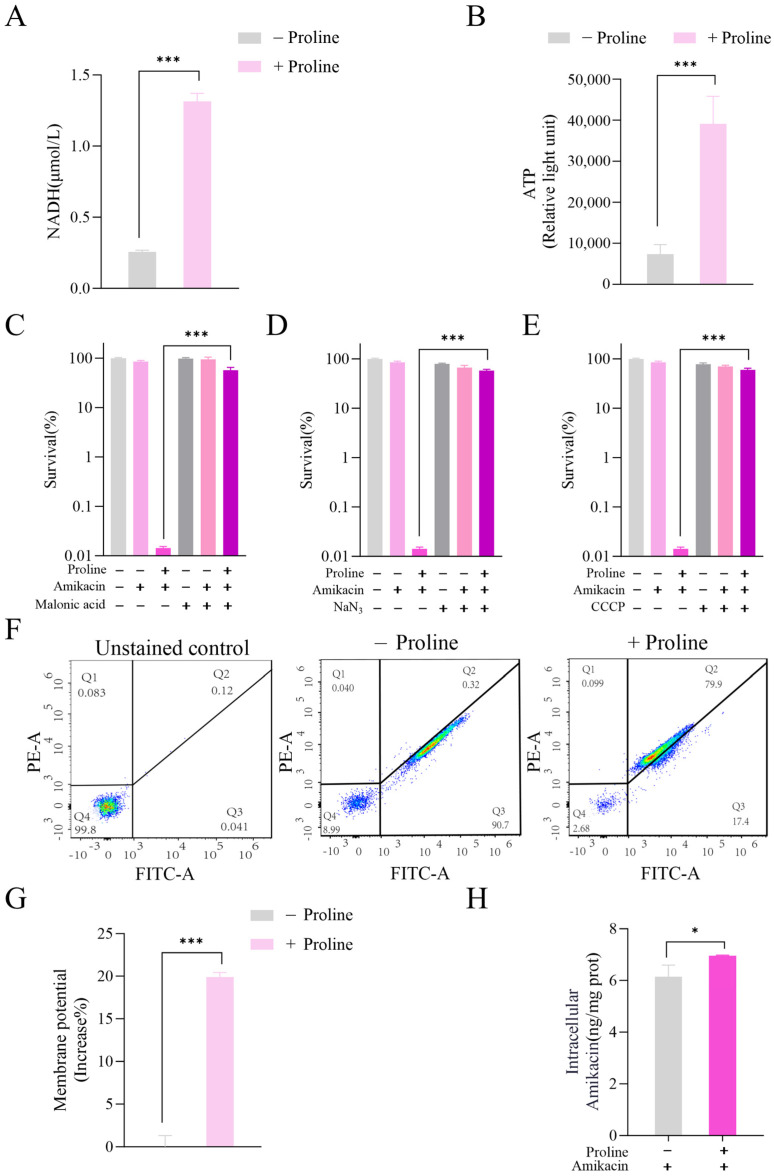
Proline enhances amikacin bactericidal activity through PMF activation and antibiotic uptake. (**A**) Intracellular NADH levels in *S. aureus* ATCC6538 treated with or without 5 mM proline. (**B**) Intracellular ATP levels in cells treated with or without 5 mM proline. (**C**–**E**) Effects of respiratory chain inhibitors on the synergistic bactericidal activity of 5 mM proline and 300 μg/mL amikacin: (**C**) 10 mM malonic acid, (**D**) 10 mM sodium azide (NaN_3_), and (**E**) 2 μM CCCP. (**F**) Flow cytometric analysis of bacterial membrane potential using DiOC_2_(3) staining. The gating strategy is shown: Q1, debris/noise; Q2, hyperpolarized cells (high Δψ); Q3, depolarized cells (low Δψ); Q4, unstained cells. The percentage of cells in each quadrant is indicated. (**G**) Effect of 5 mM proline on membrane potential (Δψ). (**H**) Intracellular accumulation of amikacin in cells treated with 300 μg/mL amikacin in the presence or absence of 5 mM proline. All data were presented as the mean ± SEM (*n* = 3). Statistical significance was determined by one-way ANOVA (* *p* < 0.05, *** *p* < 0.001).

The bactericidal activity of aminoglycoside antibiotics is associated with proton motive force (PMF) [48,49]. We observed that exogenous supplementation of proline significantly increased NADH content (Figure 3A) and intracellular ATP levels (Figure 3B) in *S. aureus*. Specific metabolic inhibitors were used: malonic acid (a competitive inhibitor of succinate dehydrogenase that blocks the conversion of succinate to fumarate in the TCA cycle and electron transport chain); sodium azide (an inhibitor of cytochrome c oxidase that disrupts terminal electron transfer in the aerobic respiratory chain), and carbonyl cyanide m-chlorophenyl hydrazone (CCCP, a protonophore that dissipates the transmembrane proton gradient). Notably, co-treatment with any of these inhibitors significantly reversed the bactericidal effect of the proline/amikacin combination, restoring bacterial survival to levels comparable to the control (Figure 3C–E). This indicates that an intact electron transport chain is essential for the survival of *S. aureus*. Further analysis found that exogenous proline significantly enhanced PMF (Figure 3F,G). As expected, proline supplementation also led to a marked increase in the intracellular accumulation of amikacin (Figure 3H). These findings demonstrate that proline potentiates the bactericidal effect by activating bacterial energy metabolism and enhancing PMF, thereby promoting the uptake of the antibiotic.

### 3.4. The Increase in Reactive Oxygen Species Levels Triggered by Proline Contributes to Its Synergistic Bactericidal Effect

**Figure 4:**
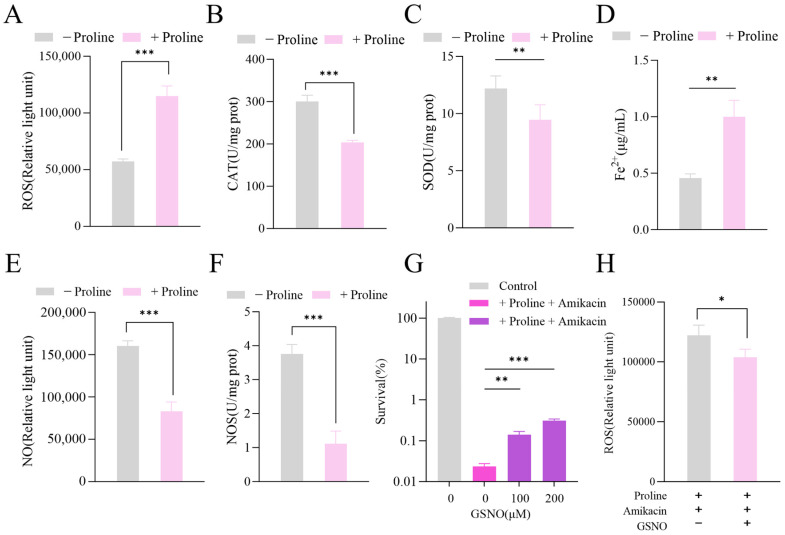
Proline potentiates amikacin killing by elevating reactive oxygen species (ROS) levels and reducing nitric oxide (NO) levels. (**A**) Intracellular ROS levels in *S. aureus* ATCC6538 treated with or without 5 mM proline. (**B**,**C**) Activities of superoxide dismutase (SOD) and catalase (CAT) in cells treated with or without 5 mM proline. (**D**) Intracellular ferrous iron (Fe^2+^) levels in cells treated with or without 5 mM proline. (**E**) Intracellular NO levels in cells treated with or without 5 mM proline. (**F**) Activities of nitric oxide synthase (NOS) in cells treated with or without 5 mM proline. (**G**) Effect of the NO donor S-nitrosoglutathione (GSNO) on the synergistic bactericidal activity of 5 mM proline and 300 μg/mL amikacin. (**H**) Intracellular ROS levels in cells treated with 5 mM proline and 300 μg/mL amikacin in the presence or absence of 200 μM GSNO. All data were presented as the mean ± SEM (*n* = 3). Statistical significance was determined by one-way ANOVA (* *p* < 0.05, ** *p* < 0.01, *** *p* < 0.001).

Antibiotic-induced bacterial killing is often linked to reactive oxygen species (ROS) generation, and exogenous metabolites can modulate ROS levels [50]. In this study, we found that exogenous proline supplementation significantly increased intracellular ROS in *S. aureus* compared to untreated controls (Figure 4A). Proline treatment also reduced the activity of key ROS-scavenging enzymes, superoxide dismutase (SOD) and catalase (CAT) (Figure 4B,C), and elevated intracellular Fe^2+^ levels (Figure 4D). Previous reports suggest that exogenous alanine can reduce intracellular nitric oxide (NO) level in other bacteria [51]. As NO plays important roles in bacterial antioxidant defense and signaling [52,53,54], we measured its levels in *S. aureus* and found that proline significantly reduced intracellular NO (Figure 4E). Further analysis of nitric oxide synthase (NOS) enzyme activity found that proline treatment also markedly suppressed NOS activity (Figure 4F), indicating that proline attenuates NO synthesis at the enzymatic level. Supplementing with the NO donor S-nitrosoglutathione reversed both the bactericidal effect of the proline/amikacin combination (Figure 4G) and the associated increase in ROS (Figure 4H). These results indicate that proline enhances amikacin killing by reducing NO levels and suppressing SOD/CAT activity.

### 3.5. Broad-Spectrum Synergy of Proline as an Aminoglycoside Potentiator and Its Mechanism

**Figure 5:**
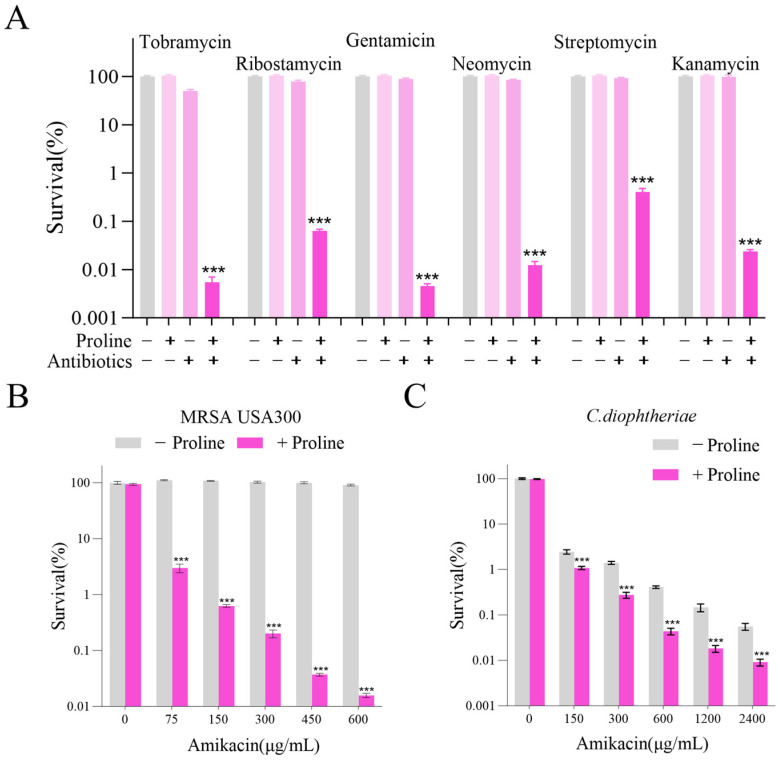
Broad-spectrum synergistic activity of proline. (**A**) Survival of *S. aureus* ATCC6538 treated with 300 μg/mL of various aminoglycosides in combination with or without 5 mM proline. (**B**,**C**) Survival of MRSA USA300 and *Corynebacterium diphtheriae* treated with 5 mM proline and increasing concentrations of amikacin. All data were presented as the mean ± SEM (*n* = 3). Statistical significance was determined by one-way ANOVA (*** *p* < 0.001).

To assess whether the synergy extends to other aminoglycosides, we tested proline in combination with a panel of such antibiotics. Beyond its synergy with amikacin, proline enhanced the bactericidal activity of multiple other aminoglycosides, including tobramycin, ribostamycin, gentamicin, neomycin, streptomycin, and kanamycin (Figure 5A). Furthermore, proline restored amikacin susceptibility in other clinically relevant Gram-positive pathogens, including the MRSA strain USA300 and *Corynebacterium diphtheriae* (Figure 5B,C). The underlying mechanism likely involves a dual action: proline enhances amikacin uptake by activating the electron transport chain and increasing the PMF. Concurrently, it elevates intracellular ROS levels by suppressing SOD and CAT activity, reducing NO levels, and activating the Fenton reaction via increased Fe^2+^. Therefore, these combined effects ultimately lead to bacterial cell death.

## 4. Discussion

Recent studies have demonstrated that exogenous metabolites can modulate both antibiotic uptake and bacterial metabolism, thereby altering antibiotic susceptibility [55]. Among these, amino acids have emerged as particularly effective adjuvants for reversing antibiotic resistance. The clinically utilized amino acid formulation 18AA significantly enhances the efficacy of ceftazidime–avibactam against multidrug-resistant pathogens [36]. Similarly, L-threonine and L-glycine potentiate gentamicin activity against MRSA [56], while proline mitigates resistance evolution under ciprofloxacin pressure [57]. These approaches, collectively termed “metabolite-driven” strategies, represent a paradigm shift in combating antimicrobial resistance by targeting bacterial metabolic vulnerabilities [58,59].

Proline plays multifaceted and critical roles in bacterial physiology. Beyond its fundamental role as a protein building block, proline can be converted to glutamate via the actions of proline dehydrogenase and proline oxidase, subsequently feeding into the tricarboxylic acid (TCA) cycles to fuel energy metabolism [60]. *S. aureus* accumulates proline under high-salt stress conditions to bolster its resistance against environmental and oxidative challenges [61,62]. During infection, *S. aureus* up-regulates genes encoding proline transporters and collagenase, enabling the scavenging of host-derived proline from degraded collagen as a carbon source to drive the TCA cycle and oxidative phosphorylation, thereby sustaining the metabolic fitness of persister cells [63]. In aquaculture environments, the role of proline in significantly improving the growth performance, feed utilization, and protein synthesis of whiteleg shrimp has been well established in aquatic nutrition [64]. Study has shown that exogenous proline enhances serum-mediated killing of *Klebsiella pneumoniae* [65]. Although the role of proline in bacterial metabolism has been extensively studied, reports on exogenous proline restoring bacterial antibiotic susceptibility remain limited, particularly in the field of aquatic pathogens.

In the present study, we screened 20 proteinogenic amino acids for their ability to potentiate antibiotics against *S. aureus*. While clinically used antibiotics like vancomycin, doxycycline and cefazolin exhibited limited synergy with amino acids (Figure S1A–C), amikacin showed a striking and potent synergistic bactericidal effect in combination with proline (Figure 1A). This combination was also effective against bacterial persisters, inhibited biofilm formation, and eradicated established biofilms. The absorption of aminoglycoside antibiotics depends on the PMF [66]. Our results demonstrated that exogenous proline activated the electron transport chain and stimulated energy production, thereby enhancing the PMF. This mechanistic link was further corroborated by the observation that co-treatment with specific respiratory chain inhibitors—malonic acid, CCCP, and sodium azide—effectively reversed the bactericidal effect, confirming that proline potentiates amikacin activity by augmenting PMF-dependent antibiotic uptake. This aligns with a previous report where exogenous proline enhanced serum-mediated killing of *K. pneumoniae*, an effect also attributed to increased PMF, potentially linked to proline influx into the pyruvate cycle [65]. The principles elucidated using amikacin as a model aminoglycoside for PMF-dependent uptake can be extended to other aminoglycosides actually used in aquaculture, such as gentamicin and neomycin.

Reactive oxygen species (ROS), primarily generated as byproducts of electron transport, are known to induce oxidative stress and enhance bacterial susceptibility to antibiotics [67]. In the bacterial antioxidant defense system, superoxide dismutase (SOD) catalyzes the dismutation of superoxide anions to hydrogen peroxide (H_2_O_2_), which is subsequently decomposed by catalase (CAT) into water and oxygen [68]. Concurrently, free ferrous iron (Fe^2+^) catalyzes the Fenton reaction, converting H_2_O_2_ into the highly toxic hydroxyl radical (•OH) [69,70]. In our study, exogenous proline supplementation concurrently suppressed the activities of SOD and CAT while increasing intracellular Fe^2+^ levels. This coordinated disruption of the oxidative stress defense system resulted in elevated intracellular ROS. Enhanced oxidative stress can arise from either increased ROS production or impaired ROS scavenging. This is consistent with reports that other exogenous metabolites, such as Fe^3+^, can modulate antibiotic resistance by altering ROS levels through similar mechanisms involving antioxidant enzymes [69].

Nitric oxide (NO) plays a dual role in bacteria: it can be directly bactericidal at high concentrations, but at physiological levels, it functions as a protective signaling molecule against oxidative stress [71,72]. We found that exogenous proline significantly reduced intracellular NO levels in *S. aureus*. Notably, supplementation with the NO donor S-nitrosoglutathione (GSNO) not only reversed the bactericidal effect of the proline/amikacin combination but also attenuated the associated increase in ROS. This suggests that proline-mediated NO depletion is a key component of its synergistic mechanism. Furthermore, exogenous alanine has been shown to reduce NO in *Vibrio alginolyticus* by modulating nitric oxide synthase activity [51], indicating that different amino acids may influence NO homeostasis via distinct pathways, a notion further supported by our observation of diminished NOS enzyme activity upon proline treatment in *S. aureus*. Basal NO levels protect bacteria through several mechanisms: activating antioxidant enzymes like SOD and CAT [52,71]; inhibiting the Fenton reaction [52]; and nitrosylating terminal cytochrome oxidases to attenuate respiratory activity, PMF, and consequently, aminoglycoside uptake [73,74]. Disruption of this protective NO homeostasis by proline therefore represents a viable strategy for reversing antibiotic resistance.

**Figure 6:**
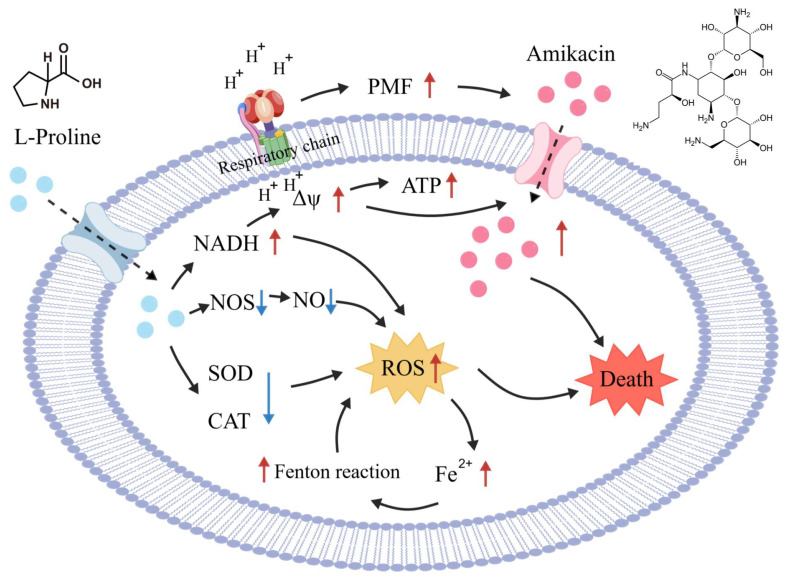
Schematic diagram of the synergistic bactericidal mechanism of action of proline in combination with amikacin. Schematic illustration of the proposed mechanism by which exogenous proline potentiates amikacin-mediated killing of *S. aureus*. Red arrows indicate upregulation, and blue arrows indicate downregulation.

This study demonstrates that exogenous proline significantly potentiates the killing of *S. aureus* by aminoglycoside antibiotics. The mechanism involves a dual action: 1. enhancing amikacin uptake by boosting electron transport chain activity and increasing PMF, 2. exacerbating oxidative stress by reducing protective intracellular NO levels through suppression of NOS enzyme activity, activating the Fenton reaction via increased Fe^2+^, and suppressing key antioxidant enzymes (SOD and CAT), ultimately leading to elevated ROS levels (Figure 6).

This study still has some limitations, which need to be further improved in the future. 1. In vivo studies: Future studies will prioritize evaluating pharmacokinetics, tissue distribution, and therapeutic efficacy in animal infection models. In vivo experiments will strengthen clinical relevance and provide robust evidence for applying this combination therapy in clinical and aquaculture settings. 2. Mechanism elucidation: Further molecular details, including proline–bacterial component interactions and NOS enzyme targets, will be explored in future work. 3. Resistance development: The potential for bacteria to develop resistance to the proline–aminoglycoside combination therapy will be systematically evaluated in future studies. 4. Metabolic and resistance heterogeneity: The metabolic pathways and resistance profiles vary among different strains. The efficacy of this combination will be validated across diverse strains in future studies. 5. Safety factors and pharmacological relevance: We have determined that the MIC of this strain against amikacin is only 1.25 μg/mL (Figure S2), while the peak clinical concentration does not exceed 25 mg/L [75]. The 300 μg/mL concentration was selected because this strain exhibits resistance in M9 medium (Figure 1B). The large concentration discrepancy likely results from differences in culture media. These results will guide dosing in subsequent in vivo studies, where doses will be adjusted to clinically achievable ranges, and host safety will be evaluated before clinical translation. 6. Environmental and nutritional impacts: The effects of in vivo nutritional and physiological factors on proline metabolism and drug activity will be addressed in future studies.

## 5. Conclusions

This study demonstrates that exogenous proline markedly potentiates the bactericidal activity of amikacin against *S. aureus*. Mechanistically, proline acts through a dual-pronged strategy: it enhances antibiotic uptake by augmenting the PMF via activation of the electron transport chain, and it simultaneously amplifies oxidative stress by elevating intracellular ROS levels. This increase in ROS is achieved through the concurrent suppression of key antioxidant enzymes (SOD and CAT), a reduction in protective NO levels mediated by decreased NOS activity, and the promotion of the Fenton reaction via increased intracellular ferrous iron. These findings found a promising strategy of using a common nutrient to reverse aminoglycoside resistance in *S. aureus*. This approach offers a novel way to combat persistent and biofilm infections and suggests a route to developing adjunctive therapies that enhance bacterial clearance while lowering antibiotic doses.

## Data Availability

The data presented in this study are available on request from the corresponding author.

## Supplementary Materials

The following supporting information can be downloaded at: https://www.mdpi.com/article/10.3390/life16071070/s1.

## References

[1] Boretti A., Banik B. (2025). Antibiotic Resistance: Revisiting Older Antibiotics for Modern Bacterial Challenges. Chem. Biodivers..

[2] Nardulli P., Ballini A., Zamparella M., De Vito D. (2023). The Role of Stakeholders’ Understandings in Emerging Antimicrobial Resistance: A One Health Approach. Microorganisms.

[3] Nass N.M., Zaher K.A. (2025). Beyond the Resistome: Molecular Insights, Emerging Therapies, and Environmental Drivers of Antibiotic Resistance. Antibiotics.

[4] FAO (2024). The State of World Fisheries and Aquaculture 2024: Blue Transformation in Action.

[5] Cabello F.C., Godfrey H.P., Tomova A., Ivanova L., Dölz H., Millanao A., Buschmann A.H. (2013). Antimicrobial Use in Aquaculture Re-Examined: Its Relevance to Antimicrobial Resistance and to Animal and Human Health. Environ. Microbiol..

[6] El-Ashker M., Monecke S., Gwida M., Rezk M., Müller E., Saad T., Akinduti P., Ehricht R. (2026). Prevalence and Genetic Characterization of Methicillin-Resistant *Staphylococcus aureus* in Commercial Aquaculture Farms in Egypt. Sci. Rep..

[7] Fri J., Njom H.A., Ateba C.N., Ndip R.N. (2020). Antibiotic Resistance and Virulence Gene Characteristics of Methicillin-Resistant *Staphylococcus aureus* (MRSA) Isolated from Healthy Edible Marine Fish. Int. J. Microbiol..

[8] Harakeh S., Yassine H., Hajjar S., El-Fadel M. (2006). Isolates of *Staphylococcus aureus* and *Saprophyticus* Resistant to Antimicrobials Isolated from the Lebanese Aquatic Environment. Mar. Pollut. Bull..

[9] Tong S.Y.C., Fowler V.G., Skalla L., Holland T.L. (2025). Management of *Staphylococcus aureus* Bacteremia: A Review. JAMA.

[10] Nazli A., Tao W., You H., He X., He Y. (2024). Treatment of MRSA Infection: Where Are We?. Curr. Med. Chem..

[11] Li J.-J., Cheng F.-S., Wei X.-J., Bai Y.-B., Wang Q., Li B., Zhou Y.-X., Zhai B.-T., Zhou X.-Z., Wang W.-W. (2025). Methicillin-Resistant *Staphylococcus aureus* (MRSA): Resistance, Prevalence, and Coping Strategies. Antibiotics.

[12] Cai W., Arias C.R. (2017). Biofilm Formation on Aquaculture Substrates by Selected Bacterial Fish Pathogens. J. Aquat. Anim. Health.

[13] Okafor U.C., Okorie-Kanu O.J., Ogugua A.J., Ikeogu C.F., Okafor S.C., Anyanwu M.U., Nwobi O.C., Anyaoha C.O., Mgbeahuruike A.C., Majesty-Alukagberie L.O. (2025). Molecular Epidemiology, Antimicrobial Resistance, and Virulence Profiles of *Staphylococcus aureus* from Fish, Aquatic Environments, and Fish Handlers in Southeast Nigeria. Microorganisms.

[14] Liu C., Bayer A., Cosgrove S.E., Daum R.S., Fridkin S.K., Gorwitz R.J., Kaplan S.L., Karchmer A.W., Levine D.P., Murray B.E. (2011). Clinical Practice Guidelines by the Infectious Diseases Society of America for the Treatment of Methicillin-Resistant *Staphylococcus aureus* Infections in Adults and Children. Clin. Infect. Dis..

[15] Bassetti M., Labate L., Melchio M., Robba C., Battaglini D., Ball L., Pelosi P., Giacobbe D.R. (2022). Current Pharmacotherapy for Methicillin-Resistant *Staphylococcus aureus* (MRSA) Pneumonia. Expert. Opin. Pharmacother..

[16] Cui K., Yang W., Liu Z., Liu G., Li D., Sun Y., He G., Ma S., Cao Y., Jiang X. (2023). Chenodeoxycholic Acid-Amikacin Combination Enhances Eradication of *Staphylococcus aureus*. Microbiol. Spectr..

[17] Park S., Lee J.-H., Kim Y.-G., Hu L., Lee J. (2022). Fatty Acids as Aminoglycoside Antibiotic Adjuvants Against *Staphylococcus aureus*. Front. Microbiol..

[18] Pavani K., Shivshetty N., Poosarla V.G., Oli A.K. (2025). Synergistic Effects of Antibiotic Combinations against *Staphylococcus aureus* in Clinical Samples from Inpatients at a Tertiary Care Facility in Hyderabad, India. Open Microbiol. J..

[19] Busari A.A., Efejene I.O., Olayemi S.O., Orororo O.C., Egbune E.O. (2024). Evaluation of Antibiotic Use and Analysis of Ciprofloxacin and Gentamicin Residue in Fish Samples from Farms in Lagos, Nigeria. Environ. Monit. Assess..

[20] Ramirez M.S., Tolmasky M.E. (2017). Amikacin: Uses, Resistance, and Prospects for Inhibition. Molecules.

[21] Zhang Z.-Y., Cao Z.-Y., Fei J., Yan B.-B., Zhang T., Fan L.-Y., Zhang W.-T., Wang C., Wang H., Su Y.-B. (2026). Fumaric Acid Restores Neomycin Efficacy against Carbapenem-Resistant *Vibrio parahaemolyticus* through Metabolic Reprogramming. J. Hazard. Mater..

[22] Zheng Y., Fu L.-H., Cao Z.-Y., Zhang T., Fei J., Jiang M., Zhou Y.-L., Shi Z., Su Y.-B. (2026). Exogenous Indole Promotes Florfenicol Tolerance in *Edwardsiella tarda*. Virulence.

[23] Peng B., Li H., Peng X.-X. (2025). Metabolic State-Driven Nutrient-Based Approach to Combat Bacterial Antibiotic Resistance. npj Antimicrob. Resist..

[24] Li X., Wu J.-Q., Long X.-R., Hu S.-B., Jiang M. (2026). Functional Proteomic Analysis Reveals *mglB*-Mediated Meropenem Resistance and Its Reversal by Galactose. Virulence.

[25] Kuang S.-F., Xiang J., Chen Y.-T., Peng X.-X., Li H., Peng B. (2024). Exogenous Pyruvate Promotes Gentamicin Uptake to Kill Antibiotic-Resistant *Vibrio alginolyticus*. Int. J. Antimicrob. Agents.

[26] Wu J.-H., Chen X.-W., Liu Y.-L., Wu J.-Y., Chen Z.-G., Peng B. (2025). Metabolism-Dependent Succinylation Governs Resource Allocation for Antibiotic Resistance. Sci. Adv..

[27] Lian L.-L., Zhang L.-S., Shen C.-G., Zhang B.-H., Zhang H.-Y., Xie Y.-Y., Lin X.-M. (2025). The Impact of Lysine Succinylation Modification of Host Factor for RNA Phage Qβ Replicase at K56 Site on the Biological Functions of *Aeromonas hydrophila*. Int. J. Biol. Macromol..

[28] Yi L.-K., Cao M.-Z., Chen X., Bai Y.-B., Wang W.-W., Wei X.-J., Shi Y.-X., Zhang Y.-Y., Ma T.-H., Zhu Z. (2024). In Vitro Antimicrobial Synergistic Activity and the Mechanism of the Combination of Naringenin and Amikacin Against Antibiotic-Resistant *Escherichia coli*. Microorganisms.

[29] Christgen S.L., Becker D.F. (2019). Role of Proline in Pathogen and Host Interactions. Antioxid. Redox Signal..

[30] Wadhawan S., Gautam S., Sharma A. (2014). Involvement of Proline Oxidase (PutA) in Programmed Cell Death of *Xanthomonas*. PLoS ONE.

[31] Zhang P.-F., Liu Z. (2024). Structural Insights into the Transporting and Catalyzing Mechanism of DltB in LTA D-Alanylation. Nat. Commun..

[32] Morawska L.P., Detert Oude Weme R.G.J., Frenzel E., Dirkzwager M., Hoffmann T., Bremer E., Kuipers O.P. (2022). Stress-Induced Activation of the Proline Biosynthetic Pathway in *Bacillus subtilis*: A Population-Wide and Single-Cell Study of the Osmotically Controlled *proHJ* Promoter. Microb. Biotechnol..

[33] Chuphal N., Malik M.A., Kishore P.S., Mohanta K.N. (2025). Amino Acids as Functional Nutrients in Stress Mitigation of Aquatic Species: Mechanisms and Applications in Aquaculture. Blue Biotechnol..

[34] Yan B.-B., Li N., Zhou Y., Kang L.-L., Dong X.-S., Xu X., An L., Meng Q.-L., Wang X.-R., Yang L. (2026). Metabolic Potentiation of Antibiotic Killing by L-Arginine in Drug-Resistant *Edwardsiella tarda*. mSystems.

[35] Peng B., Su Y.-B., Li H., Han Y., Guo C., Tian Y.-M., Peng X.-X. (2015). Exogenous Alanine and/or Glucose plus Kanamycin Kills Antibiotic-Resistant Bacteria. Cell Metab..

[36] Xiang J., Zhou Y.-Q., Yuan S.-C., Zhang X.-L., Wang S.-W., Chen Z.-G., Lin L.-R., Liu Z.-Q., Li H., Peng B. (2026). Compound Amino Acid Synergizes Ceftazidime-Avibactam to Eradicate Extracellular and Facultative Intracellular MDR Pathogens. Cell Rep..

[37] Xiang J., Tian S.-Q., Wang S.-W., Liu Y.-L., Li H., Peng B. (2024). Pyruvate Abundance Confounds Aminoglycoside Killing of Multidrug-Resistant Bacteria via Glutathione Metabolism. Research.

[38] Yan B.-B., Dong X.-S., Wang J.-P., Li X.-Y., An L., Wang X.-R., Zhang L.-G., Meng Q.-L., Wang C. (2023). Glutamate-Pantothenate Pathway Promotes Antibiotic Resistance of *Edwardsiella tarda*. Front. Microbiol..

[39] Qureshi K.A., Fahmy N.A., Parvez A., Almahasheer H., Permatasari D., Jaremko M., Abdallah E.M. (2026). Biofilms and Antimicrobial Resistance: Mechanisms, Clinical Implications, and Emerging Interventions. Chem. Biodivers..

[40] Niu H.-X., Gu J.-Y., Zhang Y. (2024). Bacterial Persisters: Molecular Mechanisms and Therapeutic Development. Signal Transduct. Target. Ther..

[41] Lu C., Zhang N., Kou S., Gao L., Peng B., Dai Y., Zheng J. (2022). Sanguinarine Synergistically Potentiates Aminoglycoside-Mediated Bacterial Killing. Microb. Biotechnol..

[42] Yang J., Zeng Z.-H., Yang M.-J., Cheng Z.-X., Peng X.-X., Li H. (2018). NaCl Promotes Antibiotic Resistance by Reducing Redox States in *Vibrio alginolyticus*. Env. Microbiol..

[43] Lu W.-J., Lu H., Wang C.-C., Wang G.-Y., Dong W.-Q., Tan C. (2023). Effectors of the Type VI Secretion System Have the Potential to Be Modified into Antimicrobial Peptides. Microbiol. Spectr..

[44] Kou T.-S., Wu J.-H., Chen X.-W., Chen Z.-G., Zheng J., Peng B. (2022). Exogenous Glycine Promotes Oxidation of Glutathione and Restores Sensitivity of Bacterial Pathogens to Serum-Induced Cell Death. Redox Biol..

[45] She P.-F., Li Z.-H., Li Y.-M., Liu S.-S., Li L.-H., Yang Y.-F., Zhou L.-Y., Wu Y. (2022). Pixantrone Sensitizes Gram-Negative Pathogens to Rifampin. Microbiol. Spectr..

[46] Keren I., Kaldalu N., Spoering A., Wang Y., Lewis K. (2004). Persister Cells and Tolerance to Antimicrobials. FEMS Microbiol. Lett..

[47] Allison K.R., Brynildsen M.P., Collins J.J. (2011). Heterogeneous Bacterial Persisters and Engineering Approaches to Eliminate Them. Curr. Opin. Microbiol..

[48] Webster C.M., Woody A.M., Fusseini S., Holmes L.G., Robinson G.K., Shepherd M. (2022). Proton Motive Force Underpins Respiration-Mediated Potentiation of Aminoglycoside Lethality in Pathogenic *Escherichia coli*. Arch. Microbiol..

[49] Webster C.M., Shepherd M. (2022). A Mini-Review: Environmental and Metabolic Factors Affecting Aminoglycoside Efficacy. World J. Microbiol. Biotechnol..

[50] Batchelder J.I., Taylor A.J., Mok W.W.K. (2024). Metabolites Augment Oxidative Stress to Sensitize Antibiotic-Tolerant *Staphylococcus aureus* to Fluoroquinolones. mBio.

[51] Kuang S.-F., Chen Y.-T., Chen J.-J., Peng X.-X., Chen Z.-G., Li H. (2021). Synergy of Alanine and Gentamicin to Reduce Nitric Oxide for Elevating Killing Efficacy to Antibiotic-Resistant *Vibrio alginolyticus*. Virulence.

[52] Gusarov I., Nudler E. (2005). NO-Mediated Cytoprotection: Instant Adaptation to Oxidative Stress in Bacteria. Proc. Natl. Acad. Sci. USA.

[53] Chaudhari S.S., Kim M., Lei S., Razvi F., Alqarzaee A.A., Hutfless E.H., Powers R., Zimmerman M.C., Fey P.D., Thomas V.C. (2017). Nitrite Derived from Endogenous Bacterial Nitric Oxide Synthase Activity Promotes Aerobic Respiration. mBio.

[54] Barraud N., Schleheck D., Klebensberger J., Webb J.S., Hassett D.J., Rice S.A., Kjelleberg S. (2009). Nitric Oxide Signaling in *Pseudomonas Aeruginosa* Biofilms Mediates Phosphodiesterase Activity, Decreased Cyclic Di-GMP Levels, and Enhanced Dispersal. J. Bacteriol..

[55] Dong Y., Liu X.-N., Xiong S.-S., Cao M.-Y., Wu H.-J., Chen L., Zhao M.-M., Zheng Y.-D., Zhang Z.-Y., Liu Y.-Y. (2025). Guanosine Enhances the Bactericidal Effect of Ceftiofur Sodium on *Streptococcus suis* by Activating Bacterial Metabolism. Virulence.

[56] Guo J., Pan Z.-Y., Fan L.-Y., Zhong Y.-L., Pang R., Su Y.-B. (2023). Effect of Three Different Amino Acids Plus Gentamicin Against Methicillin-Resistant *Staphylococcus aureus*. Infect. Drug Resist..

[57] Wang H.-P., Wu X.-J., Xu J., Lu Z.-M., Hu B.-L., Zhu L.-Z., Lu H.-J. (2025). Proline Mitigates Antibiotic Resistance Evolution Induced by Ciprofloxacin at Environmental Concentrations. J. Hazard. Mater..

[58] Pinheiro F., Warsi O., Andersson D.I., Lässig M. (2021). Metabolic Fitness Landscapes Predict the Evolution of Antibiotic Resistance. Nat. Ecol. Evol..

[59] Li X., Feng D.-Y., Zhou J.-X., Wu W.-B., Zheng W.-Z., Gan W.-L., Jiang M., Li H., Peng X.-X., Zhang T.-T. (2024). Metabolomics Method in Understanding and Sensitizing Carbapenem-Resistant *Acinetobacter baumannii* to Meropenem. ACS Infect. Dis..

[60] Mouammine A., Eich K., Frandi A., Collier J. (2018). Control of Proline Utilization by the Lrp-like Regulator PutR in *Caulobacter crescentus*. Sci. Rep..

[61] Roos G., Garcia-Pino A., Van Belle K., Brosens E., Wahni K., Vandenbussche G., Wyns L., Loris R., Messens J. (2007). The Conserved Active Site Proline Determines the Reducing Power of *Staphylococcus aureus* Thioredoxin. J. Mol. Biol..

[62] Lehman M.K., Sturd N.A., Razvi F., Wellems D.L., Carson S.D., Fey P.D. (2023). Proline Transporters ProT and PutP Are Required for *Staphylococcus aureus* Infection. PLoS Pathog..

[63] Urso A., Monk I.R., Cheng Y.-T., Predella C., Wong Fok Lung T., Theiller E.M., Boylan J., Perelman S., Baskota S.U., Moustafa A.M. (2024). *Staphylococcus aureus* Adapts to Exploit Collagen-Derived Proline during Chronic Infection. Nat. Microbiol..

[64] Zou D., Yang M., Chen Z., Lin P., Li Y., Liu X., Tan B., Ye C. (2025). Effects of Proline on Growth Performance, Protein Synthesis and Cold Resistance in White Shrimp (Litopenaeus Vannamei). Anim. Nutr..

[65] Kou T.-S., Shang Y.-Y., Zhang Q.-C., Tian S.-Q., Li J., Yang L.-N., Min L., Peng B. (2025). Exogenous Proline Promotes Serum Killing of *Klebsiella pneumoniae*. Virulence.

[66] Wei X.-Y., Gao J.-C., Zhou D.-D., Xu C.-J., Chen P., Chen S.-P., Zhang Y.-H., Liu X.-H., Li G.-X., Zhu G.-B. (2024). Murepavadin Promotes the Killing Efficacies of Aminoglycoside Antibiotics against *Pseudomonas aeruginosa* by Enhancing Membrane Potential. Antimicrob. Agents Chemother..

[67] Van Acker H., Coenye T. (2017). The Role of Reactive Oxygen Species in Antibiotic-Mediated Killing of Bacteria. Trends Microbiol..

[68] Jomova K., Alomar S.Y., Alwasel S.H., Nepovimova E., Kuca K., Valko M. (2024). Several Lines of Antioxidant Defense against Oxidative Stress: Antioxidant Enzymes, Nanomaterials with Multiple Enzyme-Mimicking Activities, and Low-Molecular-Weight Antioxidants. Arch. Toxicol..

[69] Huang Y.-F., Li Y., Chen J.-Y., Lin J.-H., Liu L., Ye J.-Z., Su Y.-B. (2022). Promoting Effect of Fe^3+^ on Gentamicin Resistance in *Escherichia coli*. Biochem. Biophys. Res. Commun..

[70] Sui X.-Y., Wang J.-C., Zhao Z.-Q., Liu B., Liu M.-M., Liu M., Shi C., Feng X.-J., Fu Y.-X., Shi D.-Y. (2024). Phenolic Compounds Induce Ferroptosis-like Death by Promoting Hydroxyl Radical Generation in the Fenton Reaction. Commun. Biol..

[71] Roberts J.M., Milo S., Metcalf D.G. (2024). Harnessing the Power of Our Immune System: The Antimicrobial and Antibiofilm Properties of Nitric Oxide. Microorganisms.

[72] Okda M., Spina S., Safaee Fakhr B., Carroll R.W. (2025). The Antimicrobial Effects of Nitric Oxide: A Narrative Review. Nitric Oxide.

[73] Gusarov I., Shatalin K., Starodubtseva M., Nudler E. (2009). Endogenous Nitric Oxide Protects Bacteria against a Wide Spectrum of Antibiotics. Science.

[74] McCollister B.D., Hoffman M., Husain M., Vázquez-Torres A. (2011). Nitric Oxide Protects Bacteria from Aminoglycosides by Blocking the Energy-Dependent Phases of Drug Uptake. Antimicrob. Agents Chemother..

[75] LSizar O., Rahman S., Sundareshan V. (2023). Amikacin. StatPearls [Internet].

