# Supplementary material for: Proline Potentiates Aminoglycoside Bactericidal Efficacy Against Staphylococcus aureus
Source: Life (Basel). 2026 Jun 26;16(7):1070. doi: 10.3390/life16071070 (PMC13413327; doi:10.3390/life16071070)
Supplement: Supplementary file 1 [file life-16-01070-s001.zip › life-4340195-supplementary.pdf]

Supplementary Materials

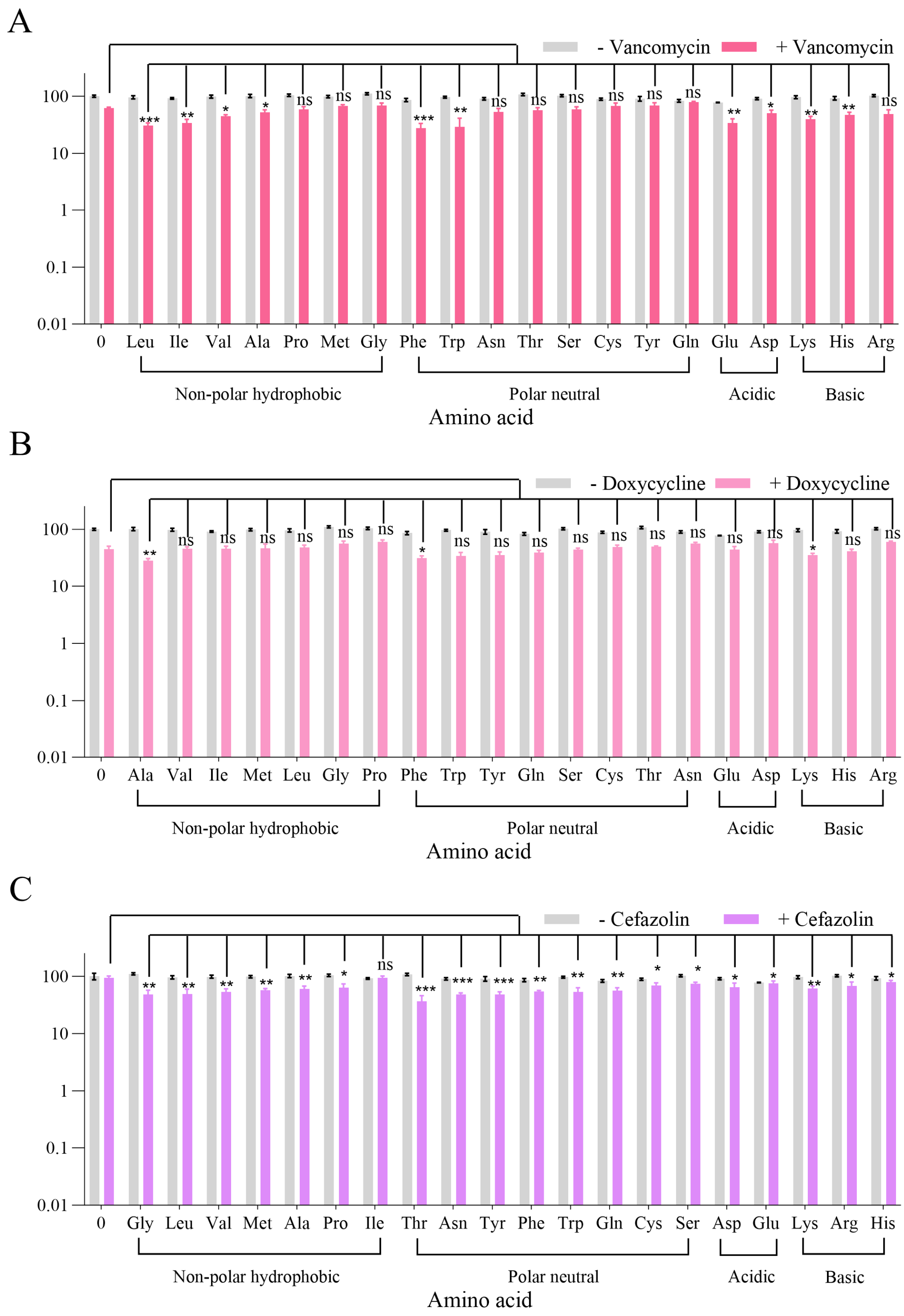

Figure S1. Synergistic bactericidal activity of various antibiotics combined with 20 amino acids. (A–C) Survival of *S. aureus* ATCC6538 treated with 300 μg/mL vancomycin (A), doxycycline (B), or cefazolin (C) in combination with 20 different amino acids (5 mM each). All data were presented as the mean ± SEM(n=3). Statistical significance was determined by one-way ANOVA (ns means no signiffcant difference, *p < 0.05, **p < 0.01, ***p < 0.001).

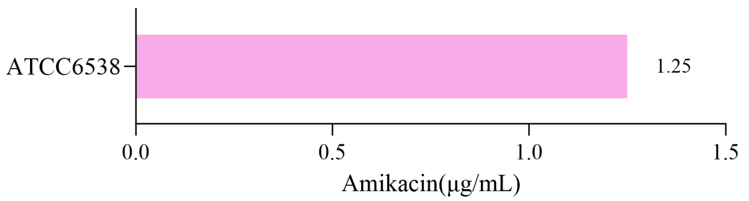

Figure S2. MIC determination of amikacin against S. aureus ATCC6538. All data were presented as the mean ± SEM(n=3).
